# Hyperinsulinemia may have a protective role in the early stages of atherosclerosis in rabbit model of hypercholesterolemia

**DOI:** 10.1186/2251-6581-11-5

**Published:** 2012-08-02

**Authors:** Shaghayegh Haghjooy Javanmard, Mehdi Nematbakhsh, Azam Feghhi, Nasim Dana

**Affiliations:** 1 Physiology Research Center, Isfahan University of Medical Sciences, Hezar jarib Avenue, Isfahan, Iran; 2 Water and electrolytes research center, Isfahan University of Medical Sciences, Isfahan, Iran

**Keywords:** Inflammation, Hypercholesterolemia, Insulin, C-reactive protein, Nitric oxide

## Abstract

**Background:**

Hypercholesterolemia causes inflammation and insulin resistance in the vasculature. Previous data suggest that vascular endothelium is a physiological target of insulin. Dyslipidemia and atherosclerosis are disorders with endothelial dysfunction that are associated with an increased production of superoxide anion, and early deficit of nitric oxide (NO) production. We examined alteration of plasma levels of insulin, C-reactive protein (CRP) and total NO metabolites (NOx), as well as fatty streak formation in the rabbit model of hypercholesterolemia.

**Methods:**

White male rabbits were fed either a high-cholesterol diet (HC; 1% cholesterol, n = 6) or control diet (c, n = 6) for one month. The serum levels of Cholesterol, LDL, HDL, NOx, insulin and CRP were measured before and after study. By the end of study, rabbits' aorta was explored for fatty streak formation.

**Results:**

The cholesterol-rich diet induced a significant increase in total cholesterol, LDL, and HDL as well as fatty streak lesions in HC group while there were no significant changes of these parameters in control group (p <0.05). There was significant difference in plasma levels of CRP, insulin and total NO metabolite between two groups of experiment. Negative significant correlation of CRP and insulin also was observed in HC rabbits (r = −0.99, p <0.05).

**Conclusion:**

Parallel NOx and insulin increment and negative correlation of CRP and insulin in HC rabbits may be suggestive a protective role of hyperinsulinemia in early atherosclerosis.

## Introduction

Considerable data suggest vascular endothelium as a physiological target of insulin [1,2]. In addition to the eminent metabolic effects, insulin has important vascular actions. Insulin stimulate production of nitric oxide (NO) from endothelium by phosphatidyl inositol 3-kinase (PI3K)-dependent signaling pathways [3]. Pathophysiologic conditions such as hyperlipidemia, and inflammation selectively impair PI3K dependent insulin signaling pathways, creating reciprocal relationships between insulin resistance and endothelial dysfunction (ED)[3], and ED is associated with deficit of NO production [4].

It is reported that NO has direct effects on the progression of atherosclerosis [5], but chronic inhibition of NO in the presence of a high-cholesterol diet has been shown to induce development of atherosclerosis [6]. Furthermore, reducing endothelial NO levels is a known mechanism for inducing insulin resistance [7]. This fact is tempting to hypothesize a potential link between insulin resistance and atherosclerosis. It has been suggested that reduced bioavailability of NO by insulin resistance may be an additional pathogenic factor in atherosclerosis [7,8]. The exact molecular relationship between insulin, ED and atherosclerosis is presently unknown [9]. In order to elucidate this relationship, studying the early variation of insulin during induction of hypercholesterolemia may help cognizance the pathogenesis of atherosclerosis.

Therefore in this study we examined plasma levels of insulin, C-reactive protein (CRP) and total NO metabolites (NOx), as well as fatty streak formation in the rabbit model of hypercholesterolemia.

## Methods

### Animals and experimental design

The study was reviewed and approved by the Ethics Committee of Isfahan University of Medical Sciences**.** Twelve white male rabbits weighing between 1.9 ± 0.1 kg were obtained from the Pasteur Institute of Iran. After 1-week acclimatization period and overnight fasting, blood samples were taken as pre-experimental sampling. Blood samples were collected in heparinated tubes, centrifuged (10,000 _ *g*), and the resulting plasma was stored at −70°C until measurement. The animals were randomized into two groups of experiments. First group (HC group) fed rabbit chow supplemented with 1% cholesterol, and the other group (control group) received rabbit chow only. All animals received food and water ad libitum. After one month of experiment, the blood samples were taken and stored again. The animals were euthanized by an overdose of sodium pentobarbital and ex-sanguinated. The animal's aortas were harvested for pathological investigation. Triglyceride, total cholesterol, high density lipoprotein (HDL) cholesterol and, low density lipoprotein (LDL) levels were measured using a standard enzymatic kit (Pars Azmoon Co, Iran)***.*** NOx measurement was done by total NO/nitrite/nitrate assay kit (R&D Systems, Minneapolis, USA) which based on the enzymatic conversion of nitrate to nitrite by nitrate reductase as previously described [10]. CRP (IBL Co, Germany) was measured using an enzyme-linked immunosorbent assay kit according to manufacturer’s instruction. Insulin was measured by the electrochemiluminescence immunoassay method using the Elecsys insulin reagents kit(Roche Diagnostics, Mannheim, Germany) on an automated Roche Modular Analytics Module E170 (Roche Diagnostics). The detection limit was 0.2 pmol/L.

### Pathologic investigation

The entire aortas, from the aortic arch to the external iliac arteries, were fixed in buffered 10% formalin for 24 h, and then embedded in paraffin. The paraffin-embedded specimens were sectioned at 5 μm. 20 sections were stained with haematoxylin and eosin, and examined by light microscopy for the fatty streak lesions formation and the intima and media thickness of aorta measurement. Then the intima / media thickness (IMT) ratios were calculated as the atheroma formation index for each animal.

### Statistical analysis

The data are reported as the mean ± SEM. A statistical software package, SPSS (version 13), was used to perform statistical analysis. The data were tested for normality and homogeneity of variance. Paired and unpaired Student's *t*-test was used to assess the significance of any changes within and between groups respectively. Statistical significance was accepted at *p <*0.05.

## Results

The cholesterol-rich diet induced a significant increase in total cholesterol, LDL-cholesterol, and HDL-cholesterol in hypercholesterolemic group while there were no significant changes of these parameters in control group. There was significant difference in total cholesterol, LDL-, and HDL between two groups of experiment (Table 1).

**Table 1:** The serum levels of total cholesterol, LDL, HDL, Insulin in two groups of the study at baseline and after experiment

	**P value(before and after)**	**After experiment**	**Before experiment**
Total cholesterol (mg/dl)			
Case	109.4 ± 12.04	2130.9 ± 171.8*	0.002
control	118.6 ± 12.1	138.02 ± 9.8	0.4
LDL-cholesterol(mg/dl)			
Case	89.2 ± 8.2	1418.9 ± 164.6*	0.003
control	86.6 ± 7.6	88.7 ± 5.1	0.07
HDL-cholesterol(mg/dl)			
Case	15.7 ± 1.4	91.7 ± 8.0*	0.019
control	13.0 ± 2.8	12.0 ± 3.4	0.6
Insulin(pmol/ml)			
Case	0.80 ± 0.06	1.16 ± 0.07*	0.04
control	0.98 ± 0.2	0.89 ± 0.2	0.1

*Significant difference between two groups of experiment (p <0.05). Student’s t-test was used to assess the significance of the parameter changes between groups. Values are mean ± S.E.

There was no significant difference in plasma level of insulin before and after experiment in control group (p <0.05) while the insulin level was significantly increased in case group (Table 1). There was significant difference in plasma levels of insulin between two groups of experiment (p <0.05) (Table 1).

**Figure 1:**
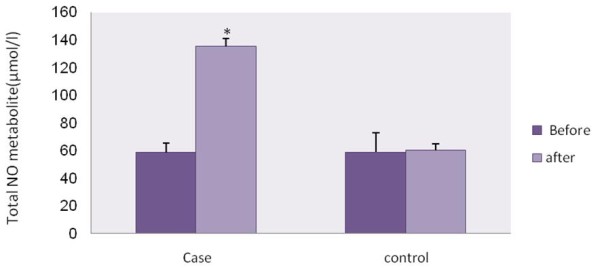
**Total nitric oxide metabolite (μmol/l) levels before and after of experiment in case and control groups.** Paired and unpaired Student’s t-test was used to assess the significance of parameter changes within and between groups respectively. (P <0.05).

Similarly, there was no significant difference in plasma level of total NO metabolite before and after experiment in control group while the total NO metabolite level was significantly increased (p <0.05) (Figure 1). There were significantly more plasma levels of total NO metabolite in cases compare to control group (p <0.05) (Figure 1).

**Figure 2:**
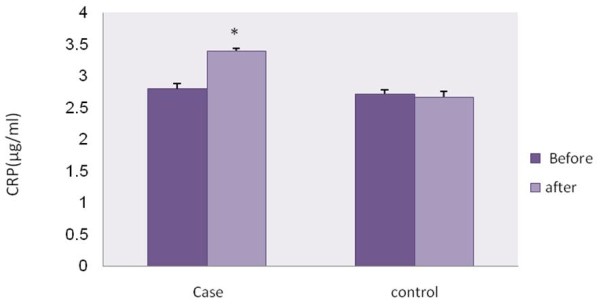
**C-reactive protein levels before and after of experiment in case and control groups.** Paired and unpaired Student’s t-test was used to assess the significance of any changes within and between groups respectively (P <0.05).

The plasma level of CRP significantly increased in case group (p <0.05) (Figure 2) whereas there was No significant difference in plasma level of CRP, insulin and total NO metabolite were found before and after experiment in control group (Figure 2). There was significant increased plasma levels of CRP in cases compare to control group (p <0.05) (Figure 2).

At the end of study, there was no fatty streak lesions in the control group aortas while the IMT ratio was 0.27 ± 0.1 in HC group (p = 0.05).

There was a significant negative correlation between plasma levels of insulin and CRP concentration (r = −0.99, p = 0.03) by the end of experiment in control group.

## Discussion

This study was designed to investigate the insulin alteration as a consequence of hypercholesterolemic diet in rabbit model of early atherosclerosis. The cholesterol-rich diet resulted in a significant increase in total cholesterol, LDL-cholesterol, and HDL-cholesterol as well as the development of early lesions that were representative of fatty streak initiation similar to those in humans. Thus, the model allowed us to examine some factor alteration during the initiation of atherosclerosis. In current experiment, CRP significantly increased in hypercholesterolemic rabbits while there was no significant change in control group.CRP has not only been proposed as risk factors of cardiovascular disease, but has also been associated with the variables of insulin resistance syndrome [11]. Since hypercholesterolemia is a major participator in the inflammatory process of atherosclerosis, several *in vitro* and *in vivo* studies have been showed the increased level of CRP in hypercholesterolemic animals and patients [12,13].

As the results of our study indicated, 30 days of cholesterol-feeding enhanced the content of total nitrite, as has been demonstrated previously [14-16]. It has been proposed that enhanced NO synthesis might be a defense mechanism to compensate for continuous inactivation of NO by oxygen-derived free radicals [17,18].Another proposed mechanisms responsible for the elevation of nitrite may be NO production by other isoforms of Nitric Oxide Synthase (NOS)enzymes. Increased NOS mRNA and protein of atherosclerotic vessels reported in other experiments showing that aortas of hypercholesterolemic rabbits release larger quantities of nitrogen oxides than do normal vessels in early atherosclerosis [19].

Interestingly, in parallel to total NO metabolite alteration, the hypercholesterolemia induced insulin increment in rabbits. At the cellular level, it has been shown that exposure to insulin increases eNOS mRNA and protein synthesis [20,21]. Furthermore, insulin increased eNOS production through increasing the activity of AP-1, a transcription factor that bind to the eNOS promoter [20].

Furthermore, in humans, infusion of insulin causes NO dependent vasodilatation and increased blood flow [22]. In both humans [23] and in animal models of insulin resistance [24], there is a specific impairment of PI3K-dependent signaling pathways. Thus, insulin resistance would be associated with a decrease in eNOS phosphorylation and decreased endothelial NO production. So, it seems that early hyperinsulinemia in the beginning of the atherosclerosis may be a protective mechanism for endothelial function. However, it has been shown that prolonged exposure of endothelial cells to high insulin levels induces a downregulation of the PI3K/Akt/eNOS axis. Such impairment of insulin signaling in prolonged hyperinsulinemia may result in ED and promote atherogenesis [25].

In this study, there was a significant negative correlation between plasma levels of insulin and CRP concentration several experiments demonstrated the anti-inflammatory effects of insulin [26-30]. It has been showed that insulin suppressed the expression of the pro-inflammatory intracellular adhesion molecule (ICAM)-1, the chemokine, monocyte chemoattractant protein-1 (MCP-1), and the key pro-inflammatory transcription factor, nuclear factor [kappa]B (NF[kappa]B) in human aortic endothelial cells at physiologically relevant concentrations [26,27]. In patients with acute myocardial infarction, insulin also suppressed C-reactive protein (CRP) and serum amyloid A (SAA) by 40% within 24 h of the start of the insulin infusion while glucose concentrations rendered unchanged [28]. This effect of insulin was corroborated in patients with myocardial infarction as well as in patients undergoing coronary artery bypass grafts in two studies [28-30].

In summary, insulin may promotes endothelial function, through increased NO production, which may has anti-inflammatory effects and cause slower atherosclerotic progression.

## Competing interests

The authors have no competing interests.

## Authors’ contributions

SHJ had substantial contributions to conception and design of the study, analysis of the data and drafting the manuscript. MN had substantial contributions to conception of the study, analysis of the data. AF did the acquisition of data and analysis. ND participated in its coordination and helped to draft the manuscript. All authors have read and approved the content of the manuscript.

## Acknowledgement

This study was supported by Isfahan University of Medical sciences, Isfahan, Iran (Grant #386327) and physiology research center.
